# Exploiting the biological response of two *Serratia fonticola* strains to the critical metals, gallium and indium

**DOI:** 10.1038/s41598-020-77447-7

**Published:** 2020-11-23

**Authors:** Joana B. Caldeira, Paula V. Morais, Rita Branco

**Affiliations:** University of Coimbra, Centre for Mechanical Engineering, Materials and Processes, Department of Life Sciences, Calçada Martim de Freitas, 3000-456 Coimbra, Portugal

**Keywords:** Biochemistry, Microbiology

## Abstract

The use of microorganisms that allows the recovery of critical high-tech elements such as gallium (Ga) and indium (In) has been considered an excellent eco-strategy. In this perspective, it is relevant to understand the strategies of Ga and In resistant strains to cope with these critical metals. This study aimed to explore the effect of these metals on two Ga/In resistant strains and to scrutinize the biological processes behind the oxidative stress in response to exposure to these critical metals. Two strains of *Serratia fonticola*, A3242 and B2A1Ga1, with high resistance to Ga and In, were submitted to metal stress and their protein profiles showed an overexpressed Superoxide Dismutase (SOD) in presence of In. Results of inhibitor-protein native gel incubations identified the overexpressed enzyme as a Fe-SOD. Both strains exhibited a huge increase of oxidative stress when exposed to indium, visible by an extreme high amount of reactive oxygen species (ROS) production. The toxicity induced by indium triggered biological mechanisms of stress control namely, the decrease in reduced glutathione/total glutathione levels and an increase in the SOD activity. The effect of gallium in cells was not so boisterous, visible only by the decrease of reduced glutathione levels. Analysis of the cellular metabolic viability revealed that each strain was affected differently by the critical metals, which could be related to the distinct metal uptakes. Strain A3242 accumulated more Ga and In in comparison to strain B2A1Ga1, and showed lower metabolic activity. Understanding the biological response of the two metal resistant strains of *S. fonticola* to stress induced by Ga and In will tackle the current gap of information related with bacteria-critical metals interactions.

## Introduction

Indium (In) and gallium (Ga) are metals belonging to group 13 on the periodic table^1^ that have diverse applications on the aerospace, telecommunication and informatics industries. Gallium is used in a wide variety of products as gallium arsenide (GaAs) or gallium nitride (GaN). These compounds are used in the manufacture of solar cells, LEDs, lasers and in the production of highly specialized circuits that are essential in high-performance computers and cell phones^2^. The main applications of indium are the production of LCDs and touch screens and it is also used in the manufacture of LEDs and some medical materials^3^. Face on the economic, industrial and technological importance of both metals and their low abundance comparatively with other elements and metals, Ga and In are included in the critical raw materials list^4^. With the increase consume of these critical metals, the continuous extraction of these metals from mine ores, secondary mineral deposits or discarded materials is necessary and consequently, the environment is often exposed to different metals resulting in environmental contamination, which disturbs the natural microbial communities of these sites^5^. However, it is known that several organisms have acquired diverse metal resistance mechanisms such as: (i) change of the metal redox state; (ii) metal cell impermeability; (iii) metals precipitation or ligation to the cell wall, (iv) uptake and intracellular chelation, (v) efflux mechanism enhancing the metal excretion, (vi) secretion of metabolites and (vii) secretion of metal chelating agents (or metabolites) to the environment^6^. Additionally, when metals enter into the cells, they often lead to an increase in reactive oxygen species (ROS) production. These species are very instable and highly reactive, which result in nucleic acids damage (mutagenic effect), protein damage (protein oxidation with loss of function) and membrane instability that compromise the membrane integrity (with degradation of the cell membrane, lipid peroxidation, and/or inhibition of electron transport chain)^7^. Therefore, organisms have developed different defence mechanisms to protect cells from these harmful effects. These mechanisms can involve activation of enzymatic antioxidants such as superoxide dismutases (SODs), catalase and peroxidases and non-enzymatic antioxidants as glutathione (GSH) and pigment production^8^. SOD is considered as first line of defence against oxidative stress by converting O^2−^ into H_2_O_2_ and catalase or/and peroxidases complete the detoxification cycle converting H_2_O_2_ to O_2_ and H_2_O^9^. In bacteria, SODs are the most important enzymes related to the cellular oxidative stress combat and are often categorized in different families based on its metallic cofactor. The first family has three SOD enzymes: Fe-SOD (SOD with iron as cofactor), Mn-SOD (with manganese) and the cambialistic SOD (which can have iron or manganese as the metallic cofactor). A second family is composed by CuZn-SOD (with copper and zinc) and is mainly found on cytosol of eukaryotic organisms. The last one is Ni-SOD (SOD with nickel) and was described on marine actinomycetes (*Actinobacteria*) and cyanobacteria^10–12^.

Many reports describe the oxidative stress promoted by heavy metals in bacteria. For example, *Rhodobacter capsulatus* and *Ochrobactrum tritici* were described to show an increase in SOD activity when the cells were exposed to tellurite and chromate, respectively^13,14^. Moreover, a SOD overexpression was also observed with *Rhodobacter sphaeroides* cells exposed to selenite^15^ and with *Proteus mirabilis* exposed to cadmium and lead^16^.

The presence of antioxidant molecules like glutathione (γ-GluCysGly, reduced form) (GSH) play a significant role in scavenging ROS in living cells. The principal reaction involving GSH is the hydrogen peroxide (H_2_O_2_) degradation to water (2GSH + H_2_O_2_ → GSSG + 2H_2_O)^17^. In bacterial cells, there are some studies relating reduced glutathione levels with resistance of *Escherichia coli* cells to oxidative stress conditions, such as heavy metals, osmotic stress and some antibiotics^18,19^. Thus, levels of GSH in cells are used as a biological parameter to evaluate the cellular oxidative stress.

Concerning the bacterial interaction with Ga and In, there is very scarce information. However, Ga seems to enter into the bacterial cells by not completely known transporters. In literature, some works reports that mutants strains (with *E. coli* and *Pseudomonas aeruginosa* strains) showed a loss of Ga resistance when genes related to iron (Fe) mechanisms were mutated^20,21^. Those works lead to the hypothesis that Fe and Ga metabolisms are related, and that Fe-metabolism can affect Ga resistance mechanisms. Another work demonstrated that Ga quenches siderophores, outside the cell, which lead to bacteria starvation and metabolic stress with the absence of Fe inside the cells^22^. Up to our knowledge, there are no studies that unveil the molecular basis of the resistance mechanisms for indium.

In this work, two *Serratia fonticola* strains isolated from metal contaminated environments were used. *S. fonticola* strains are Gram-negative bacteria from *Enterobacteriaceae* family that can be found in different environments, including drinking water, soil, sewage, humans and animals^23^. Both strains resistant to the critical metals Ga and In were selected to study the main effects of Ga and In on these resistant strains and to correlate the high resistance to these metals with the activation of biological strategies for the detoxification of the metal, namely the potential mechanisms behind the oxidative stress generated in response to the presence of these critical metals.

Since the literature related to the present subject is very scarce, this study will help to understand the effect of these two metals in bacterial cells and how bacterial strains are able to survive in presence of critical metals.

## Results

### Minimum inhibitory concentration (MIC) and minimum bactericidal concentration (MBC)

The MIC and MBC values obtained for both metals, in liquid assays, are shown in Table 1. While the MIC value for In was 0.75 mM for both strains, the MIC values for Ga were 1.5 mM and 2 mM for strains A3242 and B2A1Ga1, respectively. The MBC for In was only quantified for strain A3242, because compared to other situations, the MBC values are higher than the maximum concentration tested, 2 mM Ga and 1 mM In.

**Table 1:** MIC and MBC values obtained for the strains to indium and gallium.

Strain code	Gallium concentration (mM)		Indium concentration (mM)	
	MIC	MBC	MIC	MBC
A3242	1.50	> 2.00	0.75	> 1.00
B2A1Ga1	2.00	> 2.00	0.75	1.00

### Analysis of protein expression

**Figure 1:**
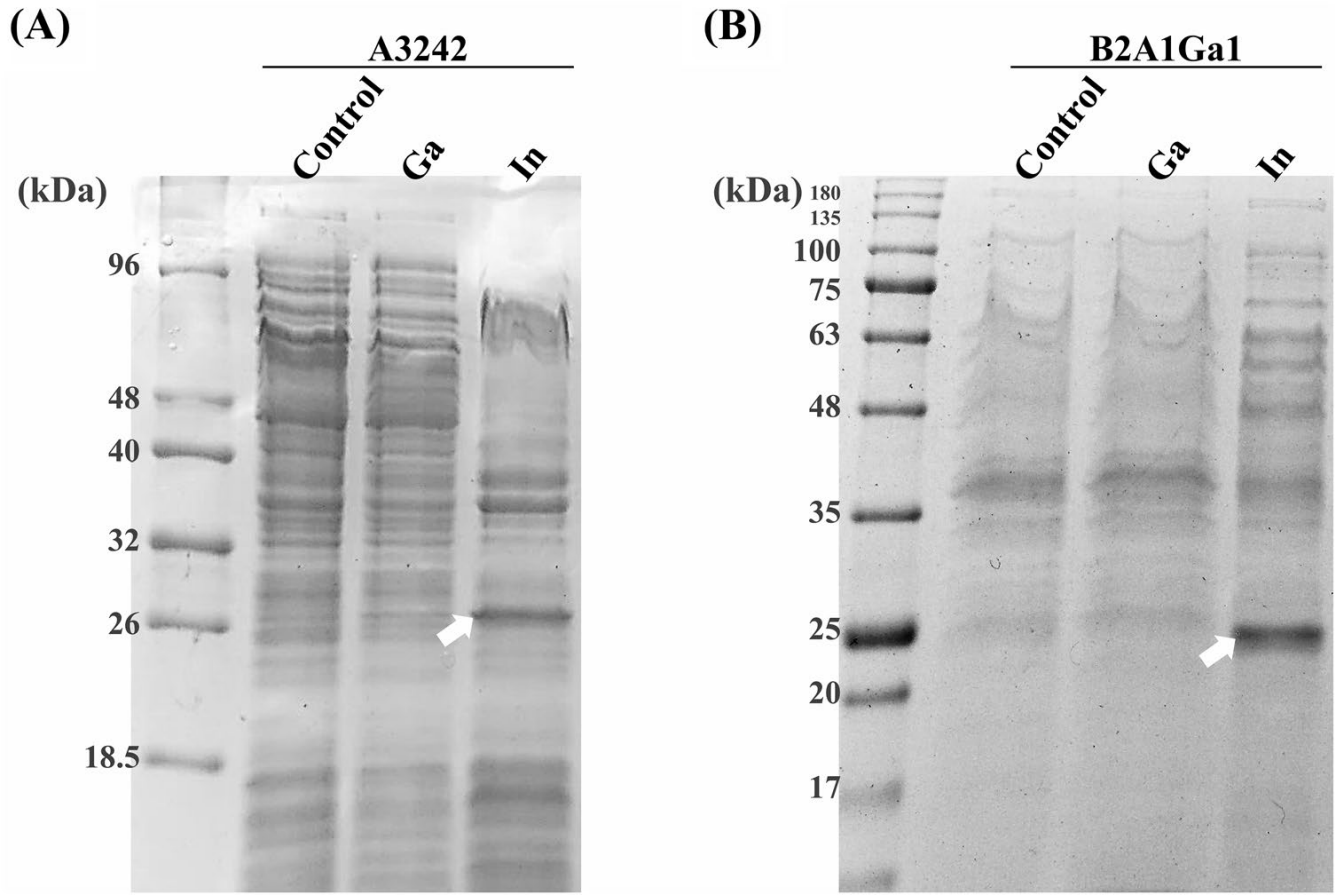
SDS-PAGE (12%) of the protein profile of bacteria grown without and with metals (0.4 mM Ga and 0.2 mM In): **(A)** M—low molecular weight protein marker (NZYTech), 1—A3242 control, 2—A3242 with Ga, 3—A3242 with In; **(B)** M—NZYColour protein marker II (NZYTech), 1—B2A1Ga1 control, 2—B2A1Ga1 with Ga, 3—B2A1Ga1 with In. The overexpressed protein band was marked with arrow.

The protein expression profile of both strains grown in presence of metals was evaluated and compared with a control situation (grown in absence of metals). Figure 1 shows that both strains, when exposed to In, overexpressed a protein with a molecular weight of approximately 25 kDa (protein band marked with an arrow). These protein bands were cut and identified by mass spectrometry with Mascot server, using the available UniProt database within the taxonomic genera *Serratia*, as belonging to iron/manganese superoxide dismutase family.

### Metal quantification

**Figure 2:**
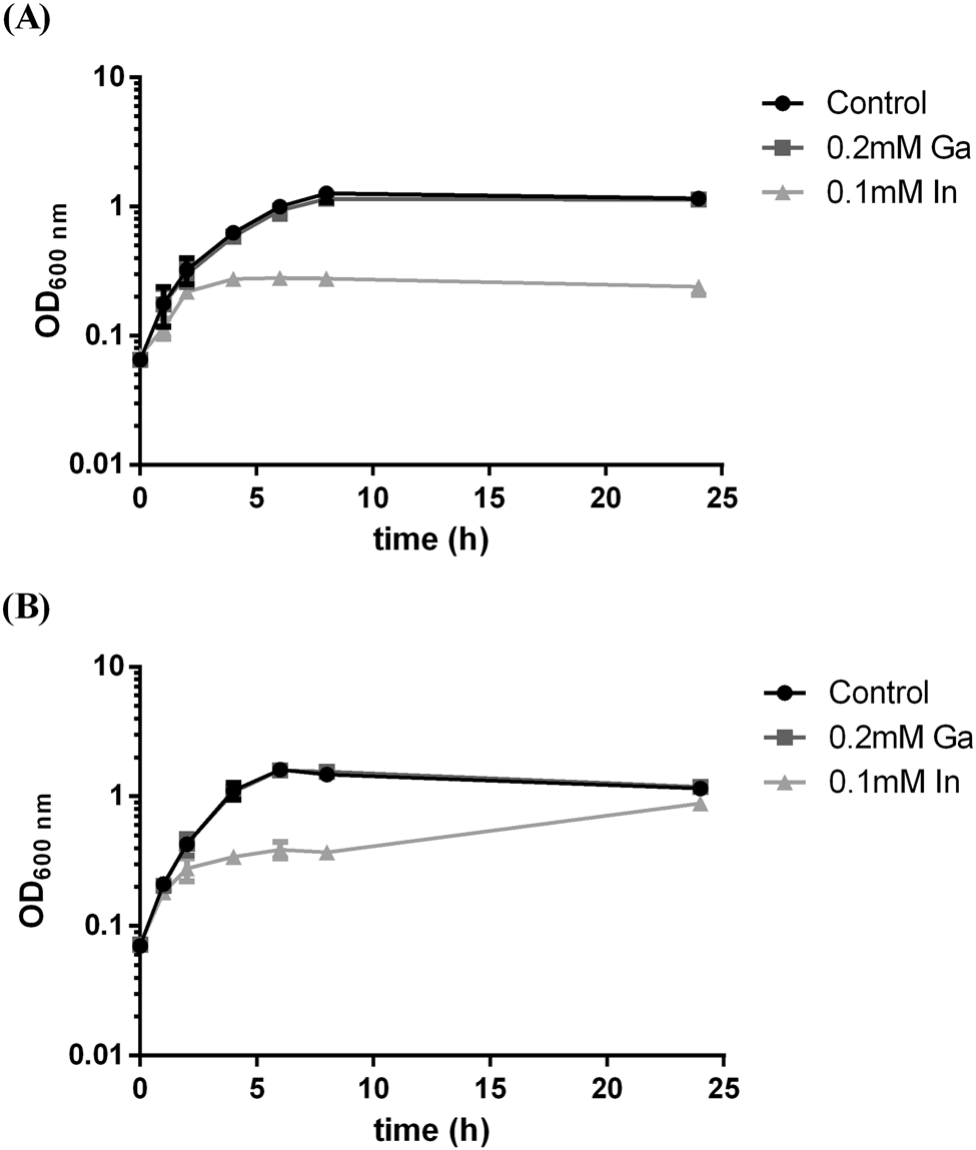
Growth curves of strains A3242 **(A)** and B2A1Ga1 **(B)** with three different conditions: control (without metal), 0.2 mM Ga and 0.1 mM In. Data shown are the mean values (± standard deviations) obtained from two independent experiments.

Growth curves of the strains in absence and presence of Ga and In were performed to show the effect of metals on bacterial growth and to select the time points to collect samples with cellular growth enough for metal quantification assays (Fig. 2). Indium had a more drastic effect in the normal growth of both strains when compared to Ga and this effect was more visible for strain A3242. Based on growth curves, the cells for metal quantification analyses were taken at two different times, 6 h of growth, corresponding to the end of exponential growth phase and 24 h, corresponding to the late stationary growth phase.

**Figure 3:**
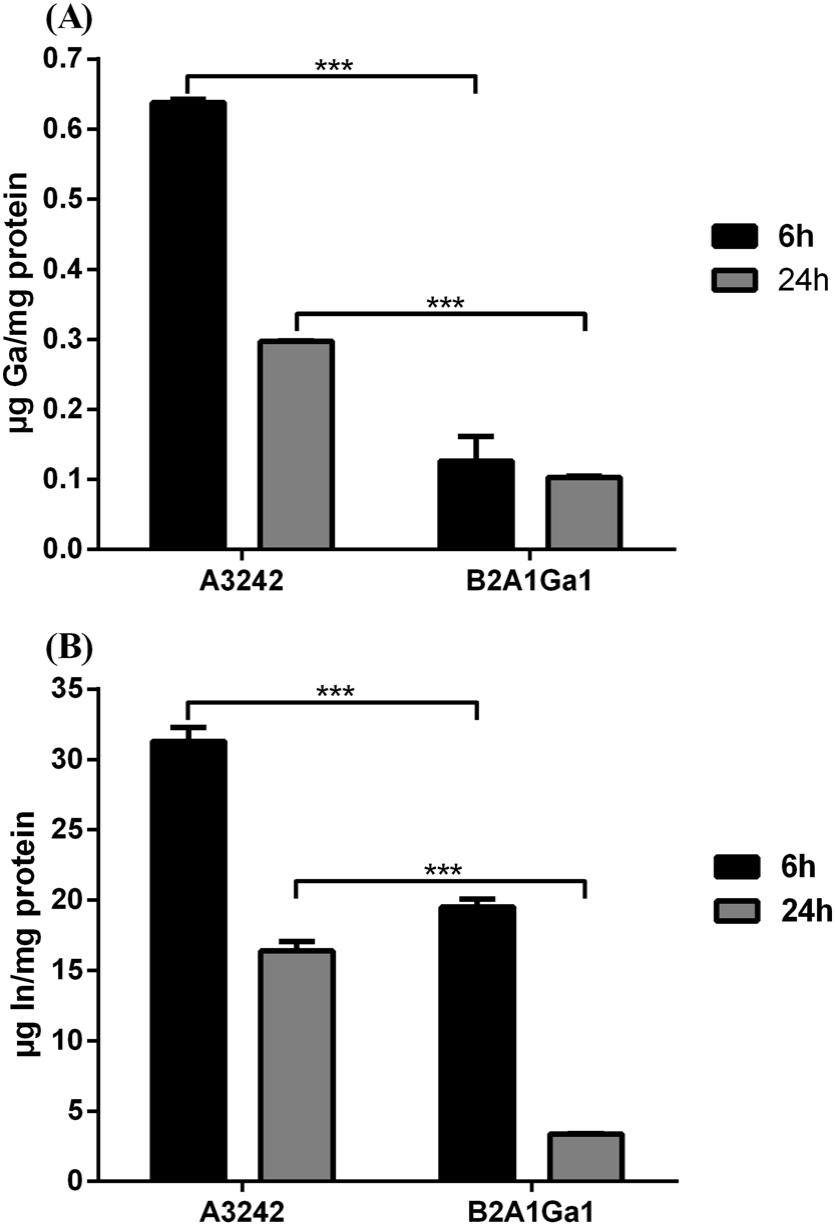
Accumulation of gallium **(A)** and indium **(B)** by strains A3242 and B2A1Ga1. Data shown are the mean values (± standard deviations) obtained from two independent experiments. ***Significantly different, p < 0.001.

The In and Ga uptake by the strains, analysed by ICP-MS, showed that strains have different levels of metal accumulation, as shown in Fig. 3. However, both strains showed higher accumulation of metals at 6 h than at 24 h of bacterial growth.

The strain A3242 showed the highest Ga levels in cells with approximately 0.64 and 0.30 µg Ga/mg protein at 6 h and 24 h, respectively, comparing with strain B2A1Ga1 that accumulated approximately 0.13 and 0.10 µg Ga/mg protein at 6 h and 24 h, respectively. In the case of In, strain A3242 also showed higher accumulation at 6 h (approximately 31.31 µg In/mg) than strain B2A1Ga1 (19.53 µg In/mg protein). At 24 h of bacterial growth, strains A3242 and B2A1Ga1 only accumulated 16.40 and 3.35 µg In/mg protein, respectively. Results suggest that metals are pumping out from the cells at the late stationary phase. Quantification of Ga in the growth medium of the best metal accumulator, strain A3232 showed values of 17,534.3 ± 253 ppm, 15,803.8 ± 8.6 ppm and 17,452 ± 35.9 ppm at 0 h, 6 h and 24 h, respectively. The In values determined in the growth medium of the strain were 7961.8 ± 65 ppm, 6395.1 ± 17.2 ppm and 6947 ± 56.1 ppm, respectively. Thus, the increase of metals in the growth medium at 24 h confirmed the release of Ga and In from cells.

### Cellular metabolic activity

**Figure 4:**
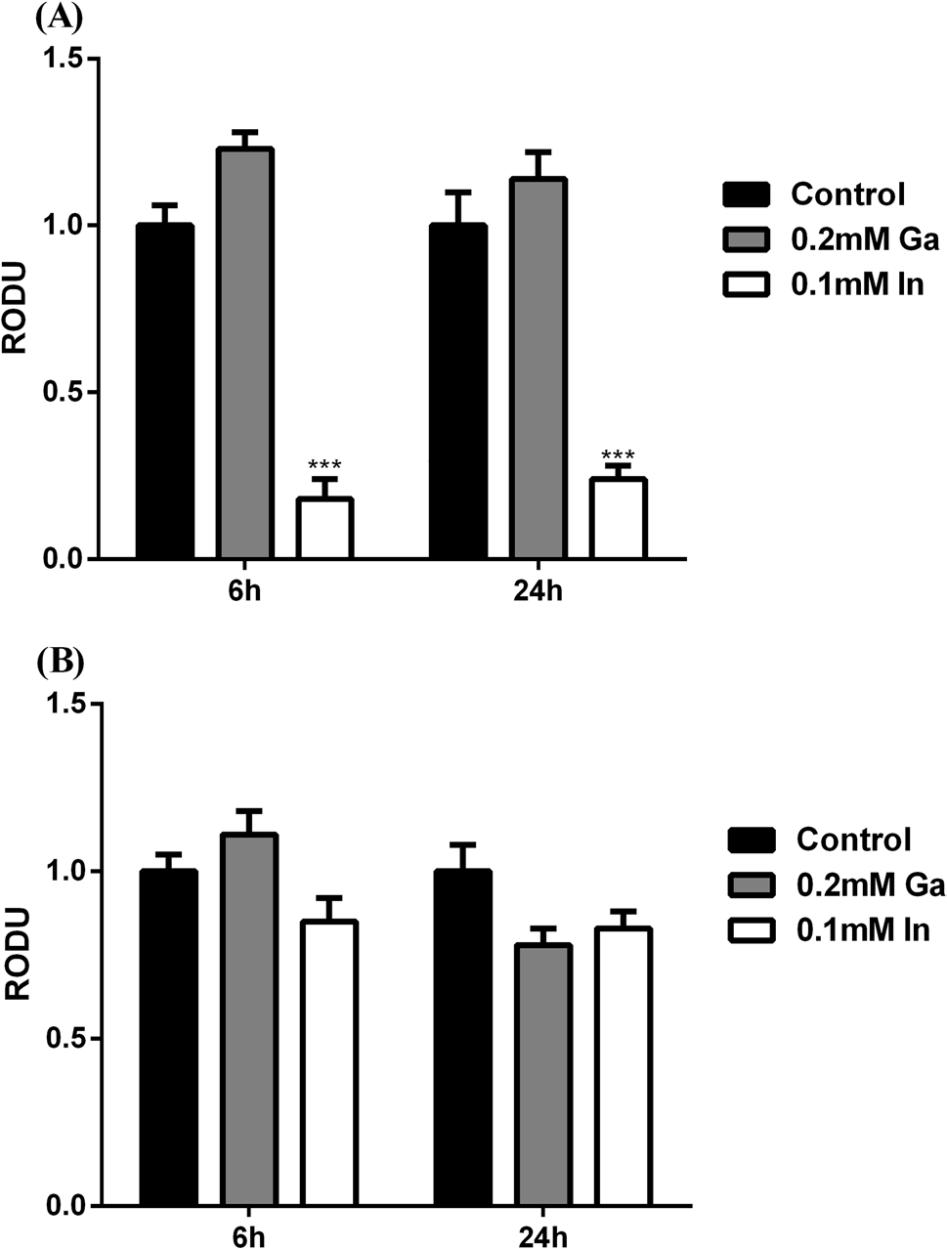
MTT assays of strains A3242 **(A)** and B2A1Ga1 **(B)**. Data shown are the mean values (± standard deviations) obtained from three independent experiments. ***Significantly different from the value of Control (without metal), p < 0.001, respectively. Relative Optical Density Units (RODU) means the ratio between the OD_550_ (absorbance at 550 nm) of the sample (test) and the OD_550_ of the control experiment.

The cellular metabolic activity was studied in both strains using the MTT assay and the results are shown in Fig. 4. Strain A3242 revealed a significant decrease of the cellular metabolic activity in the presence of In, showing activity values of 5.6 and 4.2 fold lower than the control, for samples taken at 6 h and 24 h of incubation, respectively. In the case of strain B2A1Ga1, Ga or In did not affect significantly its metabolic activity.

### ROS quantification

The oxidative stress induced by Ga and In was studied through the quantification of the intracellular ROS concentration in cells exposed to those metals and compared with control (growth without metals).

**Figure 5:**
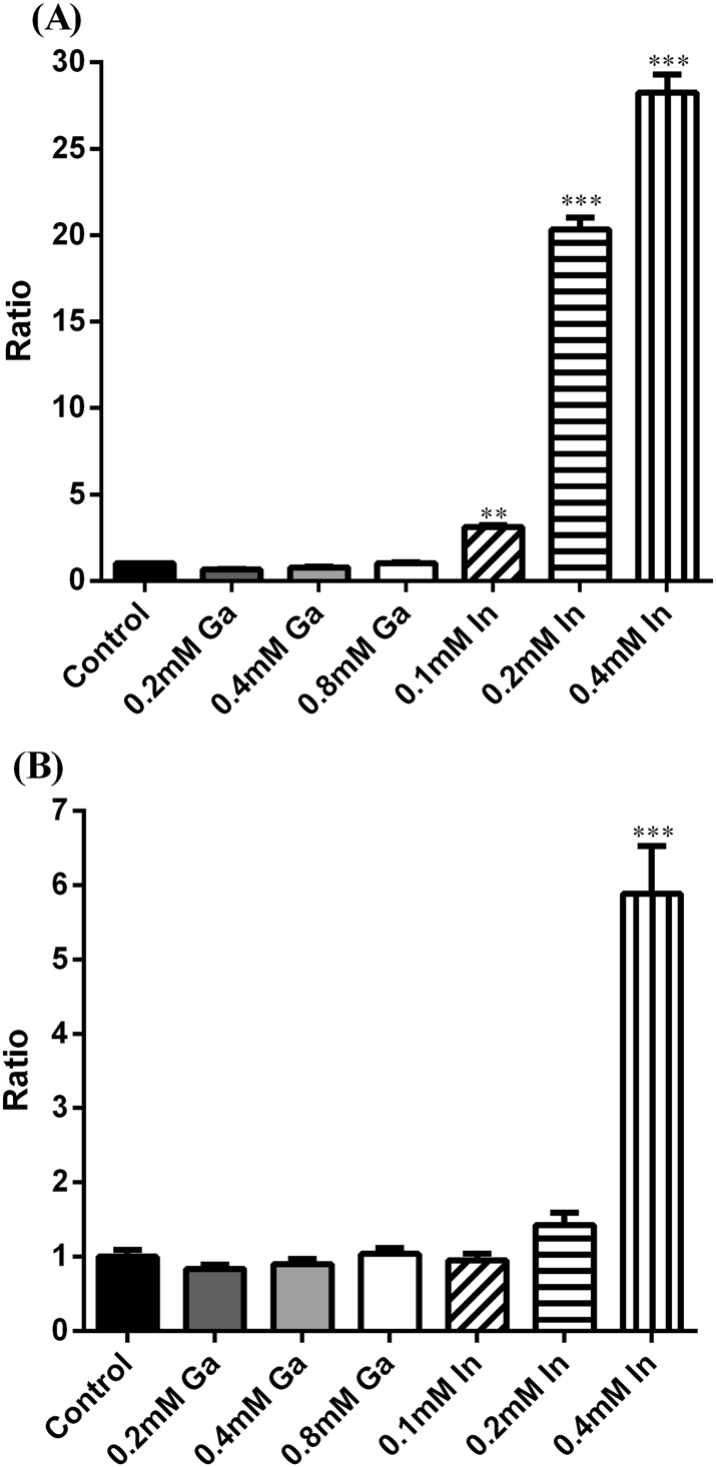
Ratio values of the relative fluorescence units (RFU of the test assay/RFU of the control assay) obtained for strains A3242 **(A)** and B2A1Ga1 **(B)**. Data shown are the mean values (± standard deviations) obtained from three independent experiments. ***Significantly different from the value of control (without metal), p < 0.001.

In Fig. 5A,B, it is possible to observe that both strains exhibited a significantly higher production of ROS in the presence of In than in the control situation, but did not show more production of ROS with Ga. The strain A3242 showed the highest values of ratio with 3.1, 20.3 and 28.3 fold higher for 0.1 mM, 0.2 mM and 0.4 mM of In, respectively. Strain B2A1Ga1 showed ratio values of 1.4 and 5.9 fold higher than control for 0.2 mM and 0.4 mM of In, respectively.

### Reduced glutathione quantification

Reduced glutathione was also quantified to confirm the potential effect of Ga and In in the oxidative stress induction. Reduced glutathione is considered an antioxidant thiol and lower concentrations of reduced glutathione (in relation with the total intracellular glutathione) mean higher oxidative stress.

**Figure 6:**
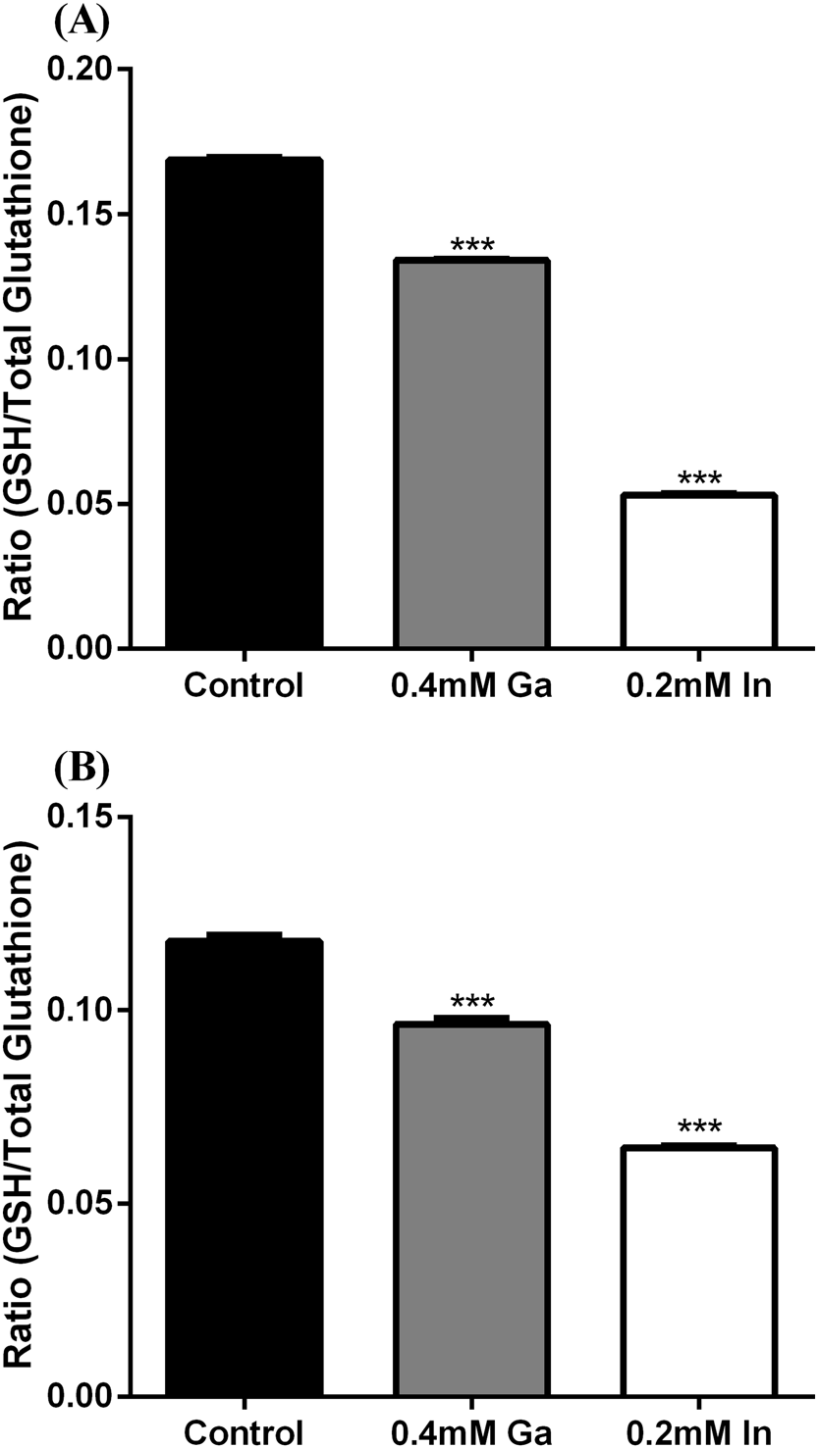
Ratio values between the reduced glutathione and the total intracellular glutathione of strains A3242 **(A)** and B2A1Ga1 **(B)**. Data shown are the mean values (± standard deviations) obtained from two independent experiments. ***Significantly different from the value of control (without metal), p < 0.001.

The Fig. 6 shows that both strains with In showed a significantly lower ratio GSH/total glutathione than the control with a decrease of 3.2 and 2.8 fold for A3242 and B2A1Ga1, respectively). In cells exposure to Ga, this ratio for both strains was also significantly lower than in control. However, the decrease of ratio GSH/total glutathione was just 1.3 and 1.2 fold of the control for A3242 and B2A1Ga1, respectively.

### SOD activity in solution

**Figure 7:**
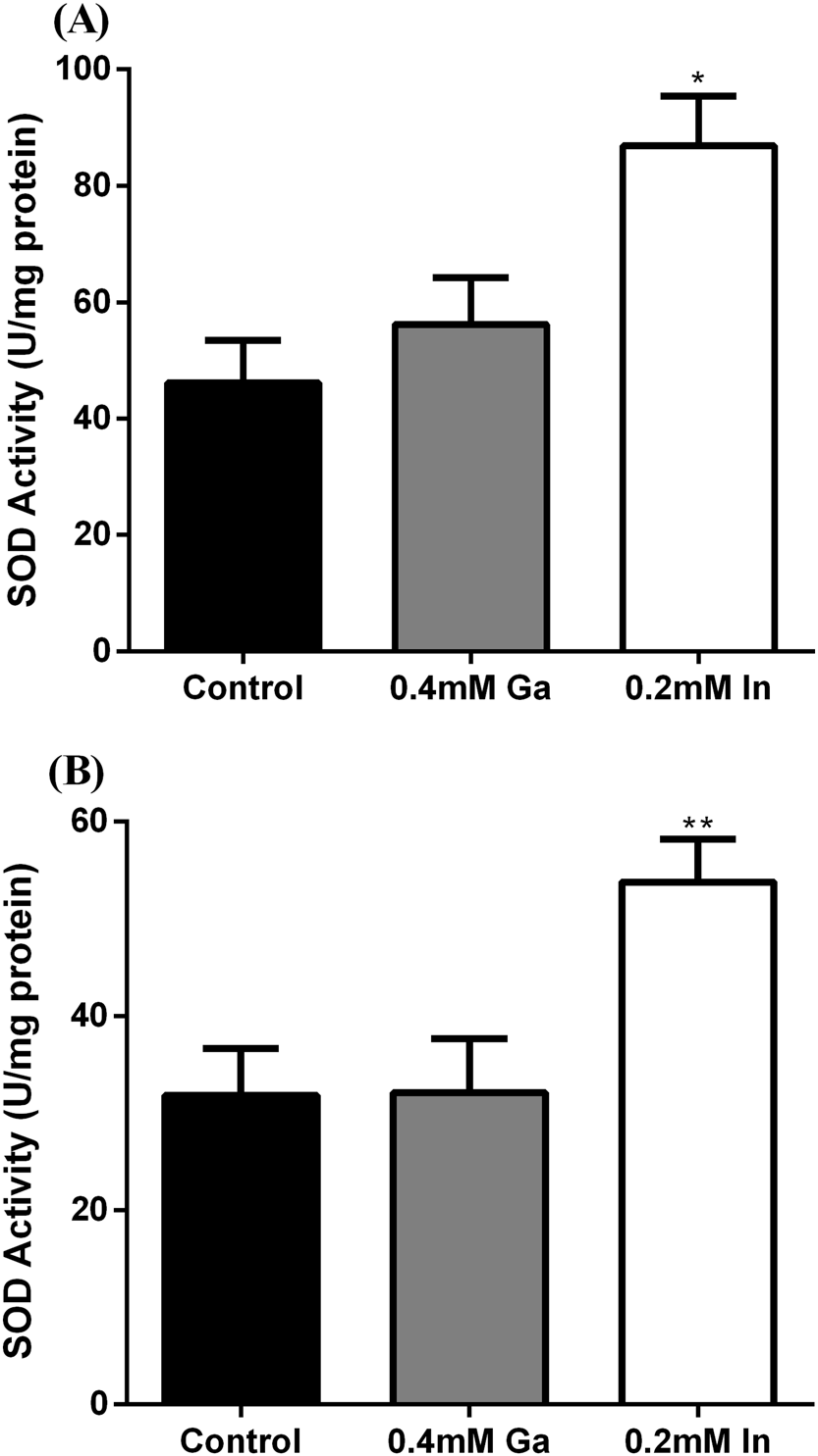
SOD activity (U/mg protein) of strains A3242 **(A)** and B2A1Ga1 **(B)**, in absence and presence of the critical metals, Data shown are the mean values (± standard deviations) obtained from two independent experiments. *,**Significantly different from the value of control (without metal), p < 0.05 and p < 0.01, respectively.

The analysis of SOD activity was performed for both strains since they showed the overexpression of a SOD enzyme in the presence of In (visible by SDS-PAGE). Figure 7 shows that both strains exhibited significantly higher SOD activity in the samples with In than in the samples with Ga or without metal. Indium exposure induced an increase of approximately twofold of SOD activity compared to the control situation without metal.

### SOD activity staining

Soluble protein samples of both strains, obtained from the growth in the presence and absence of In, were run in NBT-PAGE electrophoresis. A sample from *Escherichia coli* BL21 was used as a reference. To have a correct identification of the SOD present in the samples, different treatments of the gels were performed. Therefore, gels were incubated with two different inhibitor solutions: KCN and H_2_O_2_.

**Figure 8:**
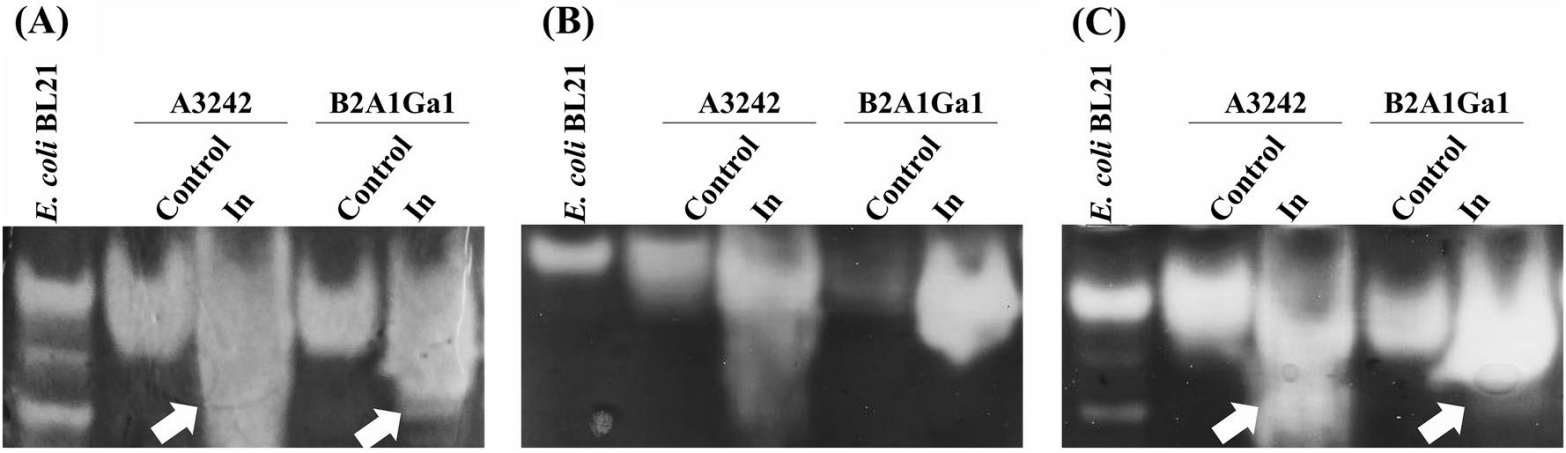
Activity staining of the protein samples electrophoresed on three independent 10% nondenatured polyacrylamide gels: **(A)** enzymatic reaction in absence of inhibitors; **(B)** enzyme incubated with 5 mM H_2_O_2_; **(C)** enzyme incubated with 5 mM KCN. Arrows indicated the additional SOD band. Original gels shown in Fig. S1.

In absence of inhibitors (Fig. 8A), protein samples of both strains from growths in the presence of In showed an additional SOD band compared to the control. When incubated with 10 mM H_2_O_2_ (Fig. 8B), an inhibitor of Fe-SOD and Cu/Zn-SOD, the lower band disappeared in In samples and in the reference strain (*E. coli* BL21). However, with 10 mM KCN (Fig. 8C), an inhibitor of Cu/Zn-SOD, the SOD bands were similar to the control gel (enzymatic reaction without inhibitor), being visible the additional lower SOD band in samples of In. These results suggest that *Serratia* strains do not have Cu/Zn-SOD and that the induced SOD by In is a Fe-SOD.

## Discussion

This work is focused on two *Serratia* strains, named A3242 and B2A1Ga1, isolated from the metal-contaminated mines of Urgeiriça and Panasqueira. Urgeiriça environment is naturally contaminated with uranium^24^ and Panasqueira with tungsten but other metals are also present in small or trace quantities^25^. Despite these sites are not reported as gallium or indium contaminated, both isolates showed extraordinary resistance to both critical metals, exhibiting high MIC values (0.75 mM for In and 1.5 mM or 2 mM for Ga) while a large number of isolates, including other tested *Serratia* strains, showed MICs of 1 mM for Ga and 0.25 mM for In as the highest values. The natural presence of metals in these environments may pressure the autochthonous bacterial communities, selecting the most resistant microorganisms to metals in general.

There are very few works exploring Bacteria-Ga/In interactions, therefore, this work aimed to understand the biological mechanisms behind oxidative stress generated in response to exposure to critical metals and its control by the cells. In this sense, these two very resistant strains were studied concerning the differential protein expression (under or overexpression) when grown with those critical metals, visible on SDS-PAGE gels. Only In induced detectable overexpression of proteins, identified as SOD enzymes. These enzymes are usually related to the control of cellular oxidative stress^9^.

The SOD activity detected in the soluble protein fractions was used to identify the additional SOD induced by In. It is known that KCN does not inhibit both Fe-SOD and Mn-SOD (inhibits CuZn-SOD) and H_2_O_2_ does not inhibit Mn-SOD but inhibits Fe-SOD and CuZn-SOD^26^. In this work, the inhibitory reactions were compared enabling the identification of the metallic cofactor present in the additional SOD enzyme. It was inhibited by H_2_O_2_ but not by KCN concluding that this enzyme was a Fe-SOD. Considering all the results observed with both SOD activity assays, it is possible to conclude that both strains have higher SOD activities when exposed to In. Previous work, relating the genome of *Serratia* sp. LCN16 strain with the high tolerance to oxidative stress predicted three genes, encoding three SOD-enzymes: two Fe/Mn-SOD (*sod2*) and one Cu/Zn-SOD (*sod1*)^27^. Up to our knowledge, currently there is no work relating the induction of SODs in *Serratia* strains with the presence of Ga and In. Nevertheless, for some strains, it was already described an overexpression of one specific SOD in the presence of heavy metals, such as overexpression of Mn-SOD with cadmium and lead in *Proteus mirabilis,* and overexpression of Fe-SOD with chromate in *Ralstonia metallidurans*^16,28^. A recent study reported a gradual and significant increase of the SOD activity in presence of antimony concentrations up to 10 mM in a strain of *Serratia marcescens* isolated from roots of the *Hedysarum pallidum* plant^29^.

It is well reported that metals are often responsible for oxidative stress in the cell (one cellular consequence from metal stress), which can affect the bacterial growth and even result in cell death^30^. Oxidative stress can be analysed using different approaches, such as ROS quantification using probes, protein damage quantification (protein carbonyl content), lipid peroxidation evaluation (malondialdehyde content), DNA damage visualization, measurement of non-enzymatic antioxidants (e.g. reduced glutathione) and assessment of enzymatic antioxidants (e.g. superoxide dismutase, catalase activities)^31^. In this work, the ROS levels quantified for both selected strains revealed that, in general, the strains showed a high ROS concentration when incubated with high concentrations of In. This is the first study to clearly show the effect of In salts in the increase of ROS production in bacterial cells. Previous works only showed the consequence of Indium Tin Oxide (ITO) nanoparticles exposure in the increase of the intracellular ROS levels on human cells^32,33^. The strains did not show a high production of ROS when exposed to high Ga concentrations, suggesting that Ga (at tested concentrations) did not induce oxidative stress in those bacteria. These results are according to the analysis of the SDS-PAGE profile. In the presence of In, both bacteria showed high oxidative stress, which resulted in high SOD activity to detoxify the cellular ROS.

The oxidative stress induced by the target critical metals was also confirmed by quantification of the reduced glutathione levels. This antioxidant exists in the reduced form in low quantities inside the cells when the cells are under oxidative stress conditions^19^. Both strains showed lower ratios (reduced glutathione/total glutathione) when subjected to the critical metals. The ratio decrease was especially relevant in the presence of In. This last result is in agreement with the ROS levels detected in cells, confirming that in *S. fonticola*, In induces high oxidative stress.

The effect of Ga and In on cellular metabolic activity was assessed by MTT assays. The decrease of activity in cells is considered a good indicator of cell redox activity^34^. The strains were affected by the exposition to both critical metals, but indium exhibited the most drastic toxic effect. Analysing the results of cellular viability, the strains, despite belonging to the same species, did not show an identical profile when exposed to the critical metals.

The two *Serratia* strains A3242 and B2A1Ga1 come from different environments and this fact can justify the notable difference in the cellular viability of these strains when grown with In. The differences between strains could also be related to different metal uptakes. The strain able to accumulate more metal showed the strongest decrease in metabolic activity. It is known that high amounts of metal inside the cells might result in many toxic effects in cells, resulting in lower cellular viability^13^. Strains showed the trend to pump metal out of cells at the late stationary phase (24 h of incubation) of growth. Metal efflux mechanisms (present or inducible) in bacteria decrease the cellular damage resulting from metal accumulation and are often responsible for bacterial metal resistance^30,35,36^. There is no description of mechanisms to pump-out In and Ga from the cells. However, considering the effect of both metals in the cellular viability, it is possible that bacteria pump-out metal in the stationary phase to limit the cellular damage (DNA, proteins, lipids). For instance, studies with *E. coli* and *Bacillus subtilis* showed that genes coding for putative manganese efflux pumps are upregulated when the bacteria are exposed to manganese^37,38^.

In conclusion, the critical metals focused on this work, exhibited different impact in two *S. fonticola* strains. Both strains when exposed to Ga just showed a significantly decrease of the ratio GSH/total glutathione, while they exhibited an increase of oxidative stress when exposed to In, with activation of biological mechanisms to control that stress, such as reduced glutathione and SOD enzymes. These strains were even able to activate an additional SOD enzyme (Fe-SOD) as a response to the toxicity induced by In. Understanding the strategies of metal resistant bacteria to cope with critical metals is important to enable and promote the use of bacteria in innovative and sustainable processes for metal recovery.

## Methods

### Bacterial strains, media and growth conditions

*S. fonticola* A3242 (isolated from Urgeiriça mine) and *S. fonticola* B2A1Ga1 (isolated from Panasqueira mine) were grown in Reasoner's 2A broth medium (R2Ab), containing per liter: 0.5 g of yeast extract, 0.5 g of proteose peptone, 0.5 g of casein, 0.5 g of glucose, 0.5 g of soluble starch, 0.3 g of K_2_HPO_4_, 0.024 g of MgSO_4_ and 0.3 g of sodium pyruvate. Bacterial growth was evaluated after incubation at 30 ºC, measuring the optical density at 600 nm (OD_600_). Metal stock solutions were prepared in a concentration of 0.5 M indium(III) chloride (InCl_3_) (Acros Organics) and 0.2 M gallium(III) nitrate (GaN_3_O_9_) (Alfa Aesar) and were sterilized by filtration.

### Minimum inhibitory concentration (MIC) and minimum bactericidal concentration (MBC) assays

The MIC of Ga and In were evaluated using the standard broth microdilution method in R2Ab^39^. Each assay was repeated in triplicate.

Additionally, the assays with metal concentrations equal and above the MIC values obtained were used to determinate the MBC. Therefore, bacterial suspensions from the wells with Ga or In concentrations that did not show bacterial growth were plated onto R2A solid medium. Bacterial growths were analysed after 48 h of incubation at 30 ºC.

### Analysis of protein expression

The bacterial resistance mechanisms to metals might be related to differential expression (under or overexpression) of specific proteins. Therefore, the protein expression profiles of cells grown in the absence (control) or presence of critical metals (0.1 mM In or 0.2 mM Ga) were evaluated by sodium dodecyl sulphate–polyacrylamide gel electrophoresis (SDS-PAGE). Firstly, the cells from 3 days of growth were centrifuged and washed twice with Phosphate Buffer Saline solution (PBS—8 g/l NaCl, 0.2 g/l KCl, 1.44 g/l Na_2_HPO_4_, 0.24 g/l KH_2_PO_4_, pH 7.4). The pellet was resuspended in 300 µl of PBS and disrupted by four cycles of 30 s pulse sonication (Sonics & Materials Inc. Danbury, Connecticut U.S.A.) at 60 A. The samples were centrifuged at 13,000 rpm (rotation per minute) for 15 min and the protein content in each fraction was quantified by Bradford method^40^. The search for a differential protein expression profile was evaluated by 0.1% sodium dodecyl sulfate (SDS)—12% polyacrylamide gel electrophoresis (PAGE), with a Coomassie Blue staining. The protein bands overexpressed in presence of the metals were identified by peptide mass spectrometry with Mascot server (Maldi-TOF, IPATIMUP, Porto).

### Metal quantification

Strains were grown in R2Ab at 30 ºC with 140 rpm spiked with 0.2 mM Ga and 0.1 mM In. Samples of bacterial growth were collected at 6 hand 24 h.

The samples were centrifuged three times at 4000 rpm for 20 min at 4 ºC, the cellular pellets were washed twice with cold PBS solution and the final bacterial pellets were lysed with an acid treatment (5% HNO_3_), heated at 50 ºC for 1 h and then centrifuged at 13,000 rpm for 10 min. The intracellular supernatants and the medium from bacterial growth at a specific time of sampling (0 h, 6 h and 24 h) were diluted for the metal quantification by Inductively Coupled Plasma Mass Spectrometry (ICP-MS)^41^. Pellets were neutralized with NaOH 0.5 M and then used to quantify the total protein by Bradford method^40^.

### Test of cellular metabolic activity

The cellular metabolic activity was evaluated using the 3-(4,5-dimethylthiazol-2-yl)-2,5-diphenyl tetrazolium bromide assay (MTT assay), according to the protocol of Wang and colaborators^42^ modified as described. Briefly, 1 ml of cellular growth, taken at the incubation times previously referred, were centrifuged, washed twice with R2Ab and the pellets were resuspended in 1 ml of medium. The cell suspensions were diluted with R2Ab to an OD_600_ of 0.2 and mixed 10:1 with MTT stock solution (5 g/ml). The mixtures were incubated with the cap tube open at 30 ºC for 1 h. After the incubation time, the mixtures were centrifuged at 10,000 *g* for 2 min and the pellets dissolved in 2.5 ml of dimethyl sulfoxide (DMSO). The samples were incubated 1 h at room temperature before quantified spectrophotometrically at 550 nm.

### ROS quantification

ROS concentration in the cells was quantified using the 2′,7′-dichlorodihydrofluorescein diacetate (H_2_DCFDA) method^43^. The protocol followed was based on the used method in a previous work^14^ and was optimized to the current assays with Ga and In. Strains grown in R2Ab, were exposed to different metal concentrations when growth reached OD_600_ of 0.2–0.3, and 2 h later, exposed to 25 μM of H_2_DCFDA for 1 h. The cells were centrifuged, washed twice with PBS and the pellets were resuspended in 1 ml PBS. The fluorescence (λ_em_ = 527 nm and λ_ex_ = 495 nm) and OD_600_ were read hourly during 5 h. ROS are determined as the Relative Fluorescence Units (RFU), obtained from the fraction between the fluorescence levels measured at λem = 527 nm and λex = 495 nm and the absorbance measured at 600 nm. The values of ratio of ROS are shown as the fraction of RFU value determined in the metal assay and the RFU value in the control situation (without metal).

### Reduced glutathione quantification

The glutathione levels in solution was quantified using the Ellman’s reagent, also known as 5,5′-dithiobis(2-nitrobenzoic acid) (DTNB) that reacts with free sulfhydryl groups in solution, using a previously described protocol^18^.

The cells were grown overnight (16 h) in R2Ab with different metal concentrations. After centrifugation, cells were washed twice with ice-cold PBS solution and the pellets were resuspended in 400 µl of 50 mM sodium phosphate, pH 7.4, (28 ml of 0.2 M NaH_2_PO_4_, 72 ml of 0.2 M Na_2_HPO_4_ and distilled water to a final volume of 400 ml) with proteases inhibitors (1 protease inhibitor cocktail tablet, EDTA-free, per 100 ml of buffer). Cells kept on ice were disrupted by four cycles of sonication (Sonics & Materials Inc. Danbury, Connecticut USA) at 60 A, with pulses of 30 s. Then, the samples were centrifuged for 1 h at 13,000 rpm to obtain soluble protein in the clear supernatant.

After protein quantification^40^, a stock for each sample was prepared with a protein concentration of 1 mg/ml (dilution prepared with 50 mM sodium phosphate buffer, pH 7.4). The assays were prepared to calculate the reduced glutathione and the total glutathione. For the first quantification, mixtures comprising sample, 6 mM DTNB and ultrapure water, 1:1:8.1, respectively, were incubated at room temperature for 6 min and the absorbance was measured at 412 nm. Total glutathione quantification assays were prepared mixing 50 µl of sample, 0.15 ml of ultrapure water, 0.7 ml of 0.3 mM NADPH, 0.1 ml of 6 mM DTNB and 10 µl of glutathione reductase solution (50 U/ml) and 6 min later the absorbance at 412 nm was measured.

### SOD activity in solution

Nitro Blue Tetrazolium (NBT) quantification method^44^ was used for quantification of SOD activity in solution. In samples with SOD, the superoxide anion (O_2_^−^) is converted to H_2_O_2_ and the reduction of NBT does not occur. Therefore, it is defined that 1 unit (U) of SOD corresponds to a decline of 50% formation the NBT photoreducted product^45^.

In this assay, cells were grown and treated as described previously for glutathione quantification experiments. The test reactions were composed by 30 µM NBT, 5 mM methionine and 26.6 µM riboflavin, all solutions prepared in SPB. For each sample, three protein concentrations were tested (10 µg, 20 µg and 30 µg protein per 1 ml of experiment). All the mixtures were subjected to illumination for 10 min with 15 W fluorescent light. After this illumination, the absorbances were read at 560 nm.

### SOD activity staining

SOD activity was also analysed by NBT–10% PAGE, with incubations in NBT-Riboflavin solutions^14^. In this assay, the cells were grown and treated as described previously for glutathione quantification experiments and analysis of SOD activity in solution. The electrophoresis was run with 18 µg of protein (each sample) at 120 V for 75 min at 4º C. The staining of the gel was based on two incubations with shaking for 20 min each, first with a staining solution of 10% riboflavin and 25% NBT and then, with a 0.1% TEMED solution. The identification of the SOD classes present in the samples was performed by adding different inhibitors solutions: 5 mM potassium cyanide solution (KCN) and 5 mM hydrogen peroxide solution (H_2_O_2_)^14^.

### Statistical analysis

Each result is indicated as the mean value of two or three independent experiments (number of independent experiments is indicated in the caption of each figure)  ±  the standard derivation. The statistical analysis of all results was performed using GraphPad Prism version 5.00 for Windows^46^, using One-way ANOVA followed by Tukey’s multiple comparisons test, except for the analysis of metal quantification (accumulation) and the cellular viability results that used Two-way ANOVA followed by Bonferroni’s multiple comparisons test.

## Supplementary information

Supplementary Figure S1.
