# Supplementary material for: Exploiting the biological response of two Serratia fonticola strains to the critical metals, gallium and indium
Source: Sci Rep. 2020 Nov 23;10:20348. doi: 10.1038/s41598-020-77447-7 (PMC7683552; doi:10.1038/s41598-020-77447-7)
Supplement: Supplementary file 1 — Supplementary Figure S1. [file 41598_2020_77447_MOESM1_ESM.pdf]

Supplementary Information

Exploiting the biological response of two *Serratia fonticola* strains to the critical metals, gallium and indium

Joana B. Caldeira[1], **Paula V. Morais**[1], **Rita Branco**[1,*]

[1] University of Coimbra, Centre for Mechanical Engineering, Materials and Processes, Department of Life Sciences, Calçada Martim de Freitas, 3000-456 Coimbra, Portugal

*Corresponding author: rbranco@uc.pt

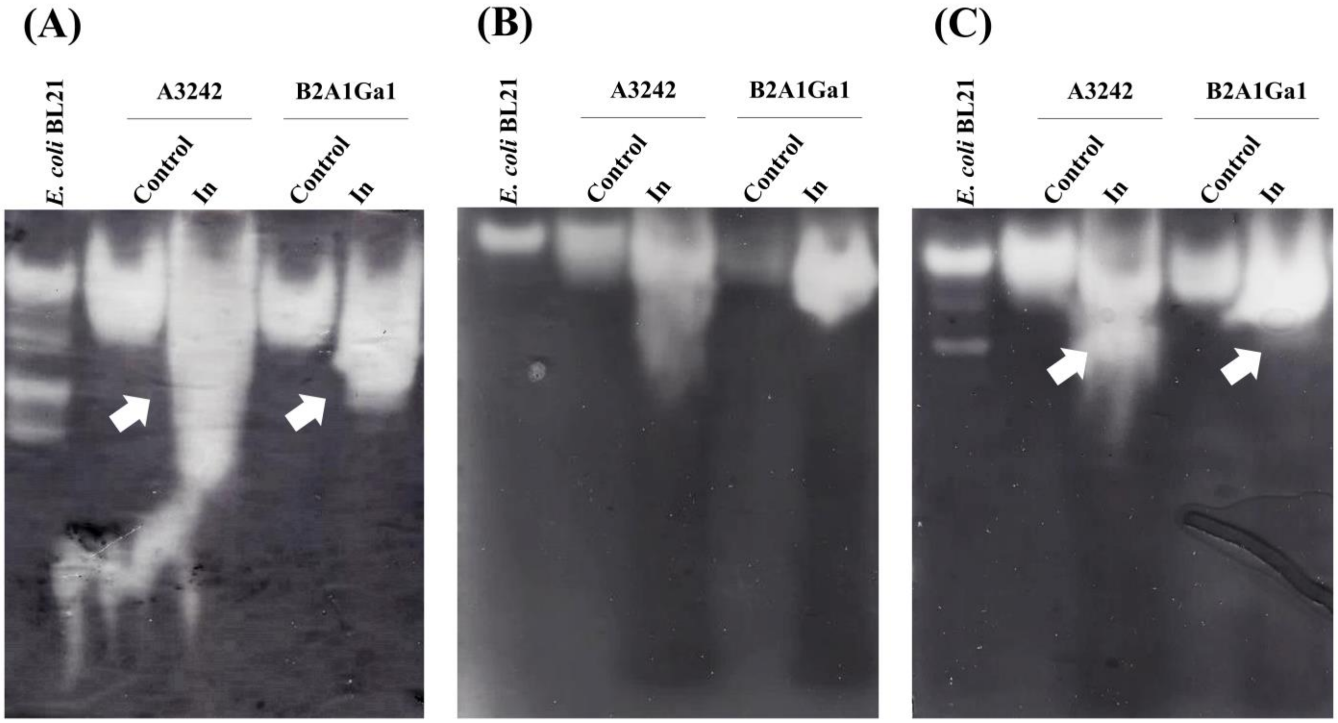

Supplementary Figure 1. The three complete gels obtained from the activity staining of the protein samples electrophoresed on 10% nondenatured polyacrylamide gel. The Figure 8 in the manuscript was cropped from this original figure. **(A)** enzymatic reaction in absence of inhibitors; **(B)** enzyme incubated with 5 mM H_2O_2; **(C)** enzyme incubated with 5 mM KCN. Arrows indicated the additional SOD band.
